# Cataract Surgery in Pet Rabbits: Clinical Presentation, Treatment, and Long-Term Outcomes

**DOI:** 10.3390/ani15192898

**Published:** 2025-10-03

**Authors:** Natthanet Sritrakoon, Kanyarat Jitsophakul, Ladawan Areevijittrakul, Aree Thayananuphat

**Affiliations:** 1 Ophthalmology Unit, Kasetsart University Veterinary Teaching Hospital, Faculty of Veterinary Medicine, Kasetsart University, Bangkok 10900, Thailand; fvetkra@ku.ac.th (K.J.); areethaya@gmail.com (A.T.); 2 Exotic Unit, Kasetsart University Veterinary Teaching Hospital, Faculty of Veterinary Medicine, Kasetsart University, Bangkok 10900, Thailand; ladawan.a@ku.th

**Keywords:** rabbit, cataract, phacoemulsification, *Encephalitozoon cuniculi*, clinical outcome

## Abstract

**Simple Summary:**

This retrospective study describes the clinical presentation, surgical management, and long-term outcomes of cataract surgery in seven pet rabbits. *Encephalitozoon cuniculi* was identified as the most common pathogen associated with cataracts (5/7 cases). All rabbits underwent phacoemulsification without intraocular lens implantation. Postoperatively, all rabbits demonstrated a positive dazzle reflex and maintained a clear visual axis with no severe complications. Owners reported improved visual behavior within two weeks. Long-term follow-up (12–40 months) indicated stable ophthalmic outcomes, with one case lost to in-person follow-up at two months but reported normal via telephone. Minor complications included lens fiber overgrowth, posterior capsular opacities, and iris synechia. These findings support the safety and efficacy of phacoemulsification for cataract management in rabbits over extended follow-up periods.

**Abstract:**

Cataracts cause vision loss in rabbits, often either spontaneously or as secondary to uveitis. This study considers the ophthalmic presentation, treatment, and outcome of phacoemulsification in seven pet rabbits: six presenting with lens cloudiness and one presenting with a white mass in the iris. Ophthalmic examinations revealed cataracts. The treatment plan was phacoemulsification. *Encephalitozoon cuniculi* was identified via an enzyme-linked immunosorbent assay technique performed on all rabbits. Ocular ultrasonography was performed to rule out retinal detachment. Phacoemulsification using the one-handed technique without intraocular lens implantation was performed in 8 of the eyes of the 7 rabbits. After surgery, the corneal wounds healed within 2 weeks. All rabbits were comfortable with opening their eyes and had a positive dazzle reflex and a clear visual axis, with no other severe complications (such as retinal detachment, intraocular hemorrhaging, or uncontrolled glaucoma) throughout the post-operative period. Postoperative complications consisted of corneal edema around the surgical wound (2 eyes; 25%); partial anterior synechiae (1 eye; 12.5%); partial posterior synechiae (5 eyes; 3 eyes before surgery and 2 eyes after surgery; 25%); posterior capsular opacities (3 eyes; 37.5%); and lens fiber overgrowths (2 eyes; 25%). In conclusion, successful phacoemulsification was achieved in the seven pet rabbits.

## 1. Introduction

Cataracts are a recognized cause of vision loss in animals, including rabbits. In a study by Innes and Williams (2018) [1], the prevalence of cataracts in rabbits was relatively low (45 out of 1000 cases). In cases of congenital cataracts, opacities were primarily located within the lens nucleus or at the level of the posterior capsule, with mean ages of 2.7 and 3.6 years, respectively. In contrast, nuclear sclerosis, an age-related change, was identified with a mean age of 6.0 years [1]. The prevalence of cataracts was reported to increase significantly in rabbits older than 8 years [2].

The etiology of cataracts in rabbits includes congenital, juvenile, age-related, inherited causes, or as secondary to uveitis [3,4]. In addition, spontaneous cataracts have been reported, particularly in New Zealand White rabbits, with the suspected cause following an autosomal recessive inheritance pattern [5]. Infection with *Encephalitozoon cuniculi* is another recognized cause of cataracts and can lead to secondary lens-induced uveitis in rabbits [6]. Ocular manifestations of *E. cuniculi* infection include spontaneous lens capsule rupture, aqueous flare, iridal granuloma, intraocular abscess, hypopyon, hypotony, synechiae formation, cataracts, and secondary glaucoma [7,8,9].

Phacoemulsification is the treatment of choice for cataract surgery [3]. Outcomes following phacoemulsification have been well-documented across various species [10,11,12,13]. However, postoperative complications are relatively common, such as postoperative ocular hypertension, capsular opacification, uveitis, retinal detachment, and glaucoma [11,12]. Long-term postoperative management and monitoring are crucial for maintaining successful visual outcomes. Despite its success in other species, reports of phacoemulsification outcomes in pet rabbits remain limited [4,6,8,14,15].

Therefore, the objective of the current study was to describe the ophthalmic presentation, surgical management, and clinical outcomes of phacoemulsification for cataract removal in pet rabbits.

## 2. Materials and Methods

### 2.1. History and Ophthalmic Examination

Six pet rabbits were presented with lens opacities (2 OD [oculus dexter; right eye], 1 OS [oculus sinister; left eye], and 3 OU [oculus uterque; both eyes]) and one rabbit was presented with a white mass at the iris OD. The age range of the rabbits (six male and one female) was 8–104 months, with a median age of 17 months. Breeds consisted of 3 Holland Lops, 3 Netherland Dwarfs, and 1 mixed breed. The duration of clinical signs reported by the owners ranged from 2 to 36 weeks, with a median duration of 12 weeks.

**Figure 1:**
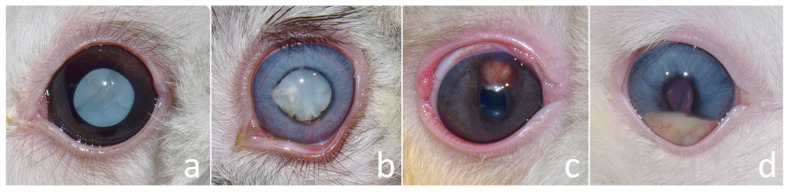
(**a**) Female Holland Lop rabbit aged 3 years presenting with mature cataract following mydriasis in left eye (OS). (**b**) Male Netherland Dwarf rabbit aged 17 months presenting with spontaneous lens capsule rupture, partial posterior synechiae, and hypermature cataract in left eye (OS). (**c**) Male Holland Lop rabbit aged 8 months presenting with iris granuloma and subcapsular cataract in right eye (OD). (**d**) Netherland Dwarf rabbit aged 1 year presenting with hypopyon, anterior synechiae, and immature cataract in right eye (OD).

Ophthalmic examination revealed cataracts (Figure 1a–c). All eyes exhibited dazzle and pupillary light reflexes, except one eye in case no. 3, which presented with a cataract and lens subluxation OD (negative dazzle reflex) and a mature cataract OS (positive dazzle reflex). The conjunctiva, cornea, anterior chamber, and iris were assessed using a slit-lamp biomicroscope (Kowa SL-17 Portable Slit-lamp Biomicroscope; Kowa Co. Ltd., Tokyo, Japan) and appeared normal in one rabbit (case no. 1). Intraocular pressures (IOPs) measured based on rebound tonometry (Icare^®^ TonoVet, Icare Finland Oy, Helsinki, Finland) ranged between 7 and 14 mmHg (within normal limits).

Other noted ocular abnormalities were uveitis (cases 2, 4, 6, 7), iritis (cases 2, 4, 5), posterior synechiae (cases 2, 4, 5), spontaneous lens capsule rupture (cases 2, 5) (Figure 1b), iridal granuloma (case 4) (Figure 1c), hypopyon (case 6) (Figure 1d), and conjunctivitis (case 2). All rabbits were comfortable opening their eyes.

The definitive diagnoses were five mature cataracts, one hypermature cataract, and two immature cataracts with phacoclastic uveitis, and two cases of iris granuloma (Table 1; Figure 1). The initial treatment plan involved phacoemulsification without intraocular lens (IOL) implantation, which was performed in one eye per rabbit, except for case no. 7, where the contralateral eye also underwent surgery subsequently.

Preoperative medical management consisted of topical anti-inflammatory therapy with 0.5% ketorolac tromethamine (Acular^®^, Allergan Pharmaceuticals Ireland, Westport, Ireland) to control lens-induced uveitis in three rabbits. In the four rabbits with active uveitis, topical 1% prednisolone acetate (Inf-Oph, Seng Thai Company, Bangkok, Thailand) administered three times daily, along with 1% atropine sulfate (Isopto^®^, Alcon, Paris, France) once daily, was prescribed until the uveitis had resolved. Subsequently, topical 0.5% ketorolac tromethamine was substituted.

**Figure 2:**
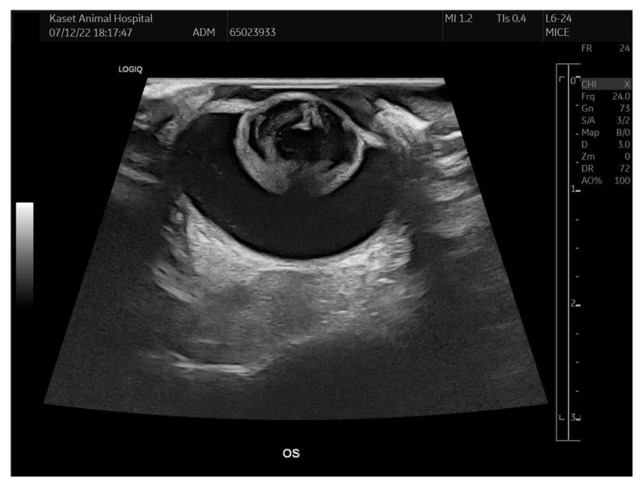
B-scan ultrasonography of eye planned for surgery, revealing cataract without evidence of retinal detachment or other intraocular abnormalities.

All rabbits underwent physical examination, hematology, and serum biochemistry testing preoperatively, with all results being within normal limits. *E. cuniculi* serology using enzyme-linked immunosorbent assay was performed, yielding five seropositive and two seronegative cases. Additionally, fenbendazole (Panacur^®^, Intervet Productions S.A., Igoville, France), at 20 mg/kg orally once daily for 28 days, was administered to all rabbits seropositive for *Encephalitozoon cuniculi*. Ocular ultrasonography (B-scan; Logiq E9, GE Healthcare, Wauwatosa, WI, USA) was performed for all surgical candidates to assess retinal integrity (Figure 2). Cataracts were confirmed without other abnormalities in most cases; however, retinal detachment was detected OD in case no. 3. Due to the presence of retinal detachment and a negative dazzle reflex, this eye was excluded from surgical consideration.

This study was approved by the Institutional Animal Care and Use Committee (IACUC) of Kasetsart University, Bangkok, Thailand (ACKU68-VET-064). This study was supervised for animal care and use for scientific research by the principal investigator, who was certified under national license No. U1-08938-2563.

### 2.2. Surgical Management

Preoperative topical medications consisted of 1% prednisolone acetate and either 0.3% ofloxacin eye drops (Exopred™, Piramal Pharma Limited, Indore, Madhya Pradesh, India) or 0.5% moxifloxacin hydrochloride (Vigamox^®^, Alcon-Couvreur NV, Puurs, Belgium) every 6 h for 3 days. Additionally, 1% atropine sulfate was administered every 8 h for 1 day before surgery (6 eyes), or 1% tropicamide (Mydriacyl^®^, Alcon-Couvreur, Puurs, Belgium) was applied every 15 min for 30 min before surgery (2 eyes) to achieve mydriasis.

Pre-anesthetic medication consisted of the intravenous administration of dexmedetomidine hydrochloride (Dexdomitor^®^, Orion Corporation, Espoo, Finland) at 0.05 mg/kg and ketamine (Ketamine-hameln, Siegfried Hameln GmbH, Hameln, Germany) at 5 mg/kg. Intravenous fluid therapy with acetated Ringer’s solution (R-cetate, General Hospital Products Public Co., Ltd., Pathum Thani, Thailand) was provided via the ear vein. Pre-oxygenation was conducted with face mask for 5 min. Before intubation, spray of larynx with lidocaine at 10 mg/puff (Xylocaine 10% spray, Aspen Bad Oldesloe GmbH, Bad Oldesloe, Germany) was administered. Endotracheal intubation was achieved using a 2.0–3.0 mm uncuffed tube. General anesthesia was maintained with 2.5–4.0% sevoflurane (Sevo^®^, Singapore Pharmawealth Lifesciences, Inc., Laguna, Philippines).

Preoperative medications consisted of subcutaneous marbofloxacin (Marbocyl^®^ 2%, Vetoquinol, Lure, France) at 2.0 mg/kg, carprofen (Rimadyl^®^, Inovat Industria Farmaceutica LTDA, Sao Paulo, Brazil) at 4.0 mg/kg or meloxicam (Metacam^®^, Boehringer Ingelheim, Mexico D.F., Mexico) at 1.0 mg/kg, and tramadol hydrochloride (Tramoda-100^®^, L.B.S. Laboratory Ltd., Bangkok, Thailand) at 4.0 mg/kg.

Standard aseptic surgical preparation involved clipping the periocular hair and flushing the conjunctival sac with a 1:50 diluted povidone–iodine solution. Local anesthesia was performed with topical 0.5% tetracaine hydrochloride eye drop (Alcon^®^, Alcon-Couvreur, Puurs, Belgium) before corneal incision. A clear corneal incision was made near the limbus at the 11 o’clock position using a slit knife (Mani^®^ Ophthalmic Knife, Mani, Inc., Tochigi, Japan). The anterior chamber was filled with 2% sodium hyaluronate (Viscovet^®^, AJL Ophthalmic, Álava, Spain) to protect the corneal endothelium.

**Figure 3:**
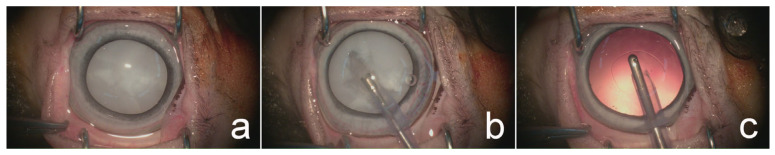
Phacoemulsification procedure for mature cataract in left eye (OS): (**a**) mature cataract with mydriatic pupil before surgery; (**b**) phacoemulsification using one-handed technique; (**c**) irrigation/aspiration of residual lens material.

**Figure 4:**
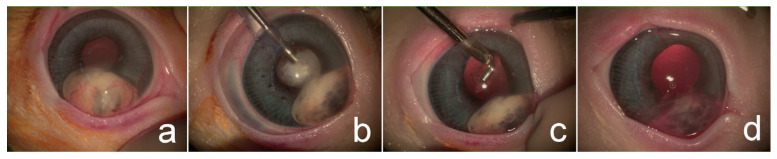
Phacoemulsification procedure for immature cataract associated with hypopyon and anterior synechiae in right eye (OD): (**a**) immature cataract with mydriatic pupil before surgery; (**b**) phacoemulsification in progress; (**c**) removal of hypopyon using irrigation/aspiration technique; (**d**) immediate postoperative appearance following cataract extraction.

A continuous curvilinear capsulorhexis was initiated with a 25-gauge needle and completed using Utrata forceps. Phacoemulsification (Centurion^®^, Alcon Surgical, Fort Worth, TX, USA) was performed using a one-handed technique without IOL implantation in the selected eye (Figure 3). Lens cortical material and iris granuloma (case no. 4), and hypopyon (case no. 6) were removed using an irrigation/aspiration handpiece (Figure 4). Sodium hyaluronate was aspirated following lens removal; subsequently, corneal incisions were closed using a simple interrupted suture pattern with 9-0 polyglycolic acid (PGA, FSSB Chirurgischenadeln GmbH, Jestetten, Germany).

The anterior chamber was reformed with balanced salt solution (BSS™, Alcon Laboratories, Fort Worth, TX, USA). Upon completion of surgery, atipamezole hydrochloride (Antisedan^®^, Orion Corporation, Espoo, Finland) at 0.5 mg/kg IV was administered to reverse dexmedetomidine sedation.

### 2.3. Postoperative Management

Postoperative oral medications consisted of marbofloxacin (Marbocyl^®^, Vetoquinol, Lure, France) at 5 mg/kg and meloxicam (Melox^®^, Siam Bheasach Co., Ltd., Bangkok, Thailand) at 0.5 mg/kg once daily for 1 week. Topical medications consisted of 1% prednisolone acetate combined with either 0.3% ofloxacin or 0.5% moxifloxacin hydrochloride every 6 h for 2 weeks, and 3 mg/mL sodium hyaluronate (Hialid^®^ 0.3, Santen Pharmaceutical Co., Ltd., Ishikawa, Japan) every 6 h for 8 weeks.

After the initial 2 weeks, topical 1% prednisolone acetate was tapered gradually, starting from every 8 h and extending over 6–8 weeks during the postoperative follow-up period.

## 3. Results

### 3.1. Postoperative Cataract Surgery

One day after surgery, all rabbits were comfortable opening their eyes. IOPs were within normal limits (7–16 mmHg) in 6 eyes, while 2 eyes exhibited hypotony (case no. 2: IOP = 4 mmHg; case no. 7 OS: IOP = 3 mmHg). Moderate conjunctivitis, mid-sized fixed pupils, and trace aqueous flare were observed in the operated eyes. Fluorescein staining was negative in all but 2 eyes (case nos. 6 and 7 OD), where superficial corneal ulcers were detected; these ulcers healed within 5 and 7 days postoperatively, respectively. Posterior capsular plaques were noted immediately postoperatively in 1 eye (case no. 3).

At two weeks postoperatively, corneal surgical wounds had completely healed, and all rabbits remained comfortable opening their eyes. IOPs were within normal limits (7–17 mmHg) throughout the follow-up period, except in one rabbit (case no. 3), which developed mild hypotony (IOP = 6 mmHg) associated with uveitis, miosis, conjunctivitis, and fibrin in the anterior chamber at week 19. This condition had resolved by week 22 following treatment with topical 1% atropine sulfate every 12 h and 1% prednisolone acetate every 8 h OS. No aqueous flare was detected during this episode. Later, at 34 months postoperatively, this rabbit exhibited a small increase in IOP (25 mmHg), which was successfully managed with a topical carbonic anhydrase inhibitor administered every 8 h, resulting in normalization of IOP.

**Figure 5:**
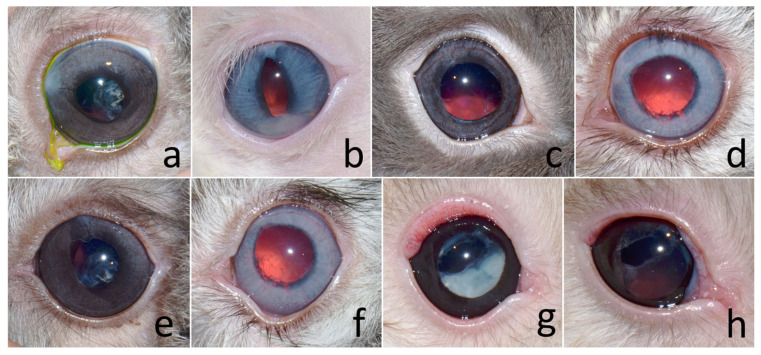
Postoperative outcomes following phacoemulsification: (**a**) corneal edema around surgical wound at 1 month postoperatively in case no. 3; (**b**) partial anterior synechiae at surgical site at 2 months in case no. 6; (**c**) partial posterior synechiae at 6 months in case no. 5; (**d**) partial posterior synechiae at 18 months in case no. 2; (**e**) posterior capsular opacity (PCO) at 3 months in case no. 3; (**f**) PCO at 21 months in case no. 2; (**g**) lens fiber overgrowth at 30 months in case no. 4; (**h**) lens fiber overgrowth at 34 months postoperatively in case no. 4.

All rabbits maintained a positive dazzle reflex and a clear visual axis throughout the postoperative period. Corneal edema around the surgical wound was observed in one eye (case no. 1) and persisted for 11 weeks postoperatively, while another eye (case no. 3) developed corneal edema two weeks after surgery, which subsequently had resolved by eight weeks (Figure 5a).

Partial anterior synechiae at the surgical site were identified in one rabbit (case no. 6) (Figure 5b). Partial posterior synechiae were observed in five rabbits: three rabbits (case nos. 2, 4, and 5) exhibited posterior synechiae due to pre-existing lens-induced uveitis, while two rabbits (case nos. 1 and 3) developed posterior synechiae secondary to mild postoperative uveitis (Figure 5c,d).

Posterior capsular opacities (PCO) were noted in three rabbits during the final follow-up evaluations (Figure 5e,f). PCO was graded according to the scoring system described by Bras et al. (2006) [16]. Two rabbits (case nos. 2 and 3) exhibited PCO grade + 1 at 21 and 3 months postoperatively, respectively, while one rabbit (case no. 4) exhibited PCO grade + 2 at 25 months postoperatively.

Lens fiber overgrowths were observed in two rabbits at 18 and 21 months postoperatively (case nos. 3 and 4, respectively) (Figure 5g,h). A summary of the postoperative complications is provided in Table 2.

The median follow-up period was 25 months (2–40 months). One rabbit (case no. 6) was lost to follow-up after 2 months; however, telephone communication with the owner indicated that no ophthalmic abnormalities were observed. All owners reported that their rabbits were alert, exhibited good visual behavior, and actively sought out objects within two weeks after surgery. Assessment of vision was shown in Table 3. All rabbits had a score of vision more than 8 points. One rabbit (case no. 1) died during the follow-up period at 12 months postoperatively, with the cause of death undetermined.

### 3.2. Control Cases

Ten rabbits were presented with cataract where the owner denied cataract surgery (control) (Table 4). The age range of the rabbits (seven male and three female) was 8 –132 months, with a median age of 77 months. Breeds consisted of 5 Holland Lops, 4 mixed breeds, and 1 Mini Rex. Ophthalmic examination revealed hypermature cataracts (7 eyes), immature cataract (6 eyes), mature cataract (4 eyes) and incipient cataract (1 eye) in the initial visiting (*n* = 18 eyes). *E. cuniculi* serology testing was performed, yielding seven seropositive and three seronegative cases. The median follow-up period was 14 months (3–51 months). The complications of cataract without surgery were uveitis (6; 33.33%), ocular hypertension (3; 16.67%), secondary glaucoma (2; 11.11%), posterior lens luxation (3; 16.67%), anterior lens luxation (2; 11.11%), lens subluxation (1; 5.56%), and posterior synechia (2; 11.11%). Severe complications eventually resulting in enucleation in case–control was 2/18 eyes (11.11%).

### 3.3. Statistical Analysis

Statistical analysis was performed using Stata Statistical Software Release 19 Version 19.5 (Stata Corp LLC, College Station, TX, USA) [17]. Descriptive statistics were used to summarize the study’s key characteristics. A chi-squared test was employed to compare the distributions of categorical variables (such as eyes, PCR results for *E. cuniculi*, and the dazzle reflex) between the cataract surgery cases and control groups, to see whether these variables were distributed differently between the two groups. Statistical significance was assessed at *p* < 0.05 (Table 5 and Table 6).

The proportion of eyes reported to be positive for *E. cuniculi* did not differ by groups, χ^2^(1, *N* = 18) = 0.1806, *p* = 0.671.

Fisher’s Exact Test was conducted to compare the proportions of eyes experiencing a positive dazzle reflex between the cataract surgery cases and the control groups. The cataract surgery cases group had 8/8 (100%) positive dazzle reflexes, while the control group had 8/18 (44.44%) positive dazzle reflexes. The two-tailed *p*-value was 0.009, indicating a statistically significant difference in positive dazzle reflexes between the groups (*p* > 0.05).

The proportion of positive dazzle reflex between *E. cuniculi* seropositive (12/18; 66.67%) and seronegative (4/8; 50%) groups was not significantly different (*p* = 0.664). However, focusing on the *E. cuniculi* seropositive, the proportion of cases with positive dazzle reflex (6/6; 100%) was more likely to be higher than that of the control group (6/12; 50%), although the difference was not statistically significant (*p* = 0.054). Likewise, in the *E. cuniculi* seronegative individuals, the proportion of cases with positive dazzle reflex (2/2; 100%) was more likely to be higher than that of the control group (2/6; 33.33%), *p* = 0.429). Management of cataract cases by operations trended to have successful outcomes. As many as 100% of cases had corrected visual acuity after surgery.

## 4. Discussion

The current study demonstrated that phacoemulsification without IOL implantation was a feasible and effective option for cataract surgery in pet rabbits, with favorable visual outcomes and a low incidence of serious complications. All rabbits in the current study retained positive dazzle reflexes and clear visual axes postoperatively, consistent with other findings that supported the visual success of cataract surgery in rabbits [6,8,14,15].

The short-term postoperative complications observed in the current study (mild conjunctivitis, transient aqueous flare, corneal ulcers, and moderate corneal edema) are common in small animal ophthalmic surgeries and are typically resolved with medical management [8,11,12,18]. Corneal edema, observed in two rabbits, was likely associated with transient endothelial dysfunction during surgery, particularly considering the shallow anterior chamber in rabbits and the lack of viscoelastic replacement in some cases [18,19]. Notably, these complications did not result in long-term vision loss or discomfort.

IOP was well maintained throughout the postoperative period in most cases, with only transient hypotony or mild ocular hypertension observed. For ocular hypertension, IOP was high, above 21–25 mmHg which can lead to optic nerve damage [20,21], whereas ocular hypotony was low IOP, generally lower than 5 mmHg, which can lead to vision-threatening complications such as corneal damage, or choroidal effusion [22]. These IOP fluctuations were consistent with other reports following cataract surgery in dogs and rabbits and are often self-limiting or medically manageable [11,12,21,23]. One rabbit exhibited ocular hypertension 34 months postoperatively, successfully managed with topical carbonic anhydrase inhibitors, reflecting the importance of long-term monitoring, even in stable cases.

Synechiae formation, both anterior and posterior, was noted in several cases. While some synechiae developed postoperatively, others were pre-existing due to lens-induced uveitis, a condition frequently associated with *Encephalitozoon cuniculi* infection [6,7,24]. Persistent posterior synechiae, despite the resolution of uveitis, reflect the chronic inflammatory nature of this condition and its potential to cause persistent adhesions [8,25].

Posterior capsular opacification (PCO), a frequent long-term complication of cataract surgery in both human and veterinary patients, was observed in three rabbits in this study [16,26,27,28,29]. The development of PCO, even in aphakic eyes, is attributed to residual lens epithelial cell proliferation and fibrosis of the posterior capsule [26,28]. Although PCO was mild in most cases in the current study and did not impair vision as assessed clinically and via owner observations, its presence supported the notion that lens capsule management plays a critical role in long-term outcomes [16,30].

Lens fiber overgrowth, noted in two cases, was consistent with other studies reporting lens regeneration potential in rabbits, particularly younger animals [31,32,33,34]. Gwon and colleagues have extensively documented lens epithelial proliferation and fiber regeneration in rabbits, especially following extracapsular extraction with an intact capsular bag [31,32]. While lens regeneration may be minimal in adult rabbits, the potential for regrowth underscores the importance of complete cortical clean-up during surgery. If the lens fiber overgrowth was developed until vision was obscured, those rabbits may need second time phacoemulsification to improve vision and IOL or capsular tension ring implantation may be needed to reduce this incidence [8].

No cases of retinal detachment, intraocular hemorrhage, phthisis bulbi, or endophthalmitis were observed, highlighting the safety of phacoemulsification in rabbits when preoperative retinal screening (such as ultrasonography) is utilized [8,25,35]. One rabbit with preoperative retinal detachment and absent dazzle reflex was excluded from surgery, reflecting the importance of thorough screening to avoid non-visual surgical candidates [35].

The limitations of this study included a small sample size, variability in follow-up periods, reliance on indirect assessment of visual function (such as dazzle reflex and owner observations), and objective methods such as obstacle course testing, rather than electroretinography (ERG). ERG was not performed in this study because it would increase the duration of anesthetic time, lead to the risk of anesthetic-related deaths in rabbits, whereas inherited retinal degeneration was not reported in pet rabbits, unlike progressive retinal atrophy which was reported in dogs [8]. However, ERG has the advantage of examining the retinal function and ruling out other retinopathies that may or may not be inherited before cataract surgery [8]. One case was lost to clinical follow-up; however, post-discharge, owner feedback indicated normal visual behavior and no recurrence of ophthalmic signs. For assessment of visual function, there was no standardized table for rating the scale of behavioral observation before, so this table requires further improvements for future use. Additionally, IOL implantation was not performed due to anatomical constraints and the current lack of commercially available rabbit-specific lenses; however, other studies have reported promising outcomes with IOLs in selected cases [4,8,15].

## 5. Conclusions

The results from the current study supported the utility of phacoemulsification as a vision-restoring procedure in pet rabbits, particularly when performed by trained surgeons using appropriate preoperative screening and postoperative care. Further research is warranted into long-term visual outcomes, optimal management of PCO, and IOL implantation in rabbits.

## Figures and Tables

**Table 1:** Signalment and ophthalmic findings of pet rabbits undergoing phacoemulsification.

Case No.	Breed	Age (Months)	Gender	Sx (Eye)	Ophthalmic Lesions	*E. cuniculi* PCR Results	Follow-Up Time (Months)
1	Mixed breed	104	Male	OS	Mature cataract OU	Negative	12
2	Netherland Dwarf	17	Male	OS	Hypermature cataract; conjunctivitis; iritis; posterior synechiae at 6 and 9 o’clock (OS)	Positive	38
3	Holland Lop	17	Male	OS	OD: Absorbing cataract with 50% lens subluxation, retinal detachment, dazzle (−), PLR (−); OS: Mature cataract, dazzle (+), PLR (−)	Negative	40
4	Holland Lop	8	Male	OD	Focal anterior lens capsular rupture; immature cataract; iritis; iridal granuloma; posterior synechiae at 12 o’clock; phacoclastic uveitis (OD)	Positive	39
5	Netherland Dwarf	20	Male	OD	Anterior lens capsule cataract; immature cataract; posterior synechiae at 2,4 and 5 o’clock; mild iritis (OD)	Positive	29
6	Netherland Dwarf	12	Male	OD	Mature cataract; focal anterior lens capsule rupture; anterior synechia at 9 o’clock; hypopyon (25%); deep corneal vascularization (OD)	Positive	2
7	Holland Lop	36	Female	OS/ OD	OS: Mature cataract; mild conjunctivitis; OD: Mature cataract	Positive	20/16

**Table 2:** Postoperative complications in pet rabbits following phacoemulsification.

Case No.	Sx (Eye)	Postoperative Complications
1	OS	Mild corneal edema (noted day 1; resolved by week 11); partial posterior synechiae (noted week 41)
2	OS	Uveitis (noted day 1; resolved by day 7); partial posterior synechiae from lens-induced uveitis (LIU) prior to surgery, increased at week 64; posterior capsular opacity (PCO) at month 21
3	OS	Posterior lens capsule plaque (immediate postoperative); mild corneal edema (noted week 2; resolved by week 8); uveitis (noted week 19; resolved by week 22); partial posterior synechiae (week 20); PCO (month 3); lens fiber overgrowth (month 21); mild ocular hypertension (IOP increase) at month 34
4	OD	Partial posterior synechiae from LIU prior to surgery, increased at week 8; PCO (month 25); lens fiber overgrowth (month 18)
5	OD	Partial posterior synechiae from LIU prior to surgery, increased at week 25
6	OD	Partial anterior synechiae at surgical site with dyscoria (noted day1); corneal ulcer (noted day 1; resolved by day 7)
7	OS	Uveitis (noted day 1; resolved by day 11); corneal ulcer (noted day 1; resolved by day 5)
	OD	None

**Table 3:** Assessment of vision in pet rabbits after phacoemulsification.

Assessment of Vision	Score of Vision (Point)		
	Always (Score = 2)	Sometimes (Score = 1)	Never (Score = 0)
No bumping any objects in house	7 eyes	1 eye	
No reluctance to move	8 eyes		
Not nervous in unfamiliar environments	8 eyes		
Can jump into the cage or step up	8 eyes		
Pass the obstacle course in exam room	7 eyes	1 eye	

**Table 4:** Signalment, ophthalmic findings and complications of ten pet rabbits which did not undergo phacoemulsification (control).

Case No.	Breed	Age (Months)	Gender	Eye	Ophthalmic Lesions	*E. cuniculi* PCR Results	Complications	Follow-Up Time (Months)
1	Holland Lop	130	Male	OU	OD: Hypermature cataract, dazzle (+); OS: Immature cataract, dazzle (+)	Positive	OD: mild ocular hypertension (IOP = 24 mmHg) and dazzle (−) at month 10	12
2	Mixed breed	132	Male	OU	OD: Immature cataract, dazzle (+); OS: Hypermature cataract, posterior lens luxation, dazzle (−)	Negative	OS: mild ocular hypertension (IOP = 23 mmHg) at month 3; OD: lens subluxation at 8–11 o’clock and dazzle (−) at month 7	9
3	Holland Lop	11	Female	OS	Focal anterior lens capsular rupture; mature cataract; iritis; iridal granuloma; complete posterior synechiae; phacoclastic uveitis; dazzle (+)	Positive	Focal anterior lens capsular rupture; mature cataract; iritis; iridal granuloma; complete posterior synechiae; phacoclastic uveitis, dazzle (+)	3
4	Mini Rex	8	Male	OS	Hypermature cataract; uveitis; complete posterior synechiae; iris bombé, dazzle (−)	Positive	Hypermature cataract; uveitis; complete posterior synechiae; iris bombé, dazzle (−)	13
5	Mixed breed	82	Female	OU	OD: Immature cataract, dazzle (+); OS: Hypermature cataract, dazzle (+)	Negative	OS: anterior lens luxation, secondary glaucoma (IOP = 98 mmHg) at month 5, dazzle (−), enucleation at month 6; OD: hypermature cataract and anterior lens luxation at month 12, corneal abscess and corneal rupture at month 27, dazzle (−), enucleation at month 27	27
6	Holland Lop	28	Male	OU	Mature cataract, dazzle (+)	Positive	OD: uveitis at month 29, dazzle (−) at month 45; OS: posterior lens luxation at month 36, uveitis at month 39, dazzle (−) at month 45	51
7	Holland Lop	60	Male	OU	Immature cataract, dazzle (+)	Positive	None	42
8	Mixed breed	88	Male	OU	OD: Immature cataract, dazzle (+); OS: Hypermature cataract, secondary glaucoma, dazzle (+)	Positive	OD: uveitis, posterior lens luxation, and dazzle (−) at month 23, secondary glaucoma at month 27; OS: uveitis and dazzle (−) at month 23, posterior lens luxation at month 26	30
9	Mixed breed	72	Male	OU	OD: Incipient cataract, dazzle (+); OS: Mature cataract, dazzle (+)	Positive	None	12
10	Holland Lop	104	Female	OU	OD: Hypermature cataract, dazzle (+); OS: Mature cataract, dazzle (+)	Negative	OS: mild ocular hypertension (IOP = 23 mmHg) at month 2, dazzle (+)	15

**Table 5:** Distribution of characters of eyes by cataract surgery cases and control groups.

Clinical Variables	Cataract Surgery Cases (*n* = 8)	Controls (*n* = 18)	*p*-Value
	Frequency (%)	Frequency (%)	
Eye			
OS	4 (50)	10 (55.56)	1.000 *
OD	4 (50)	8 (44.44)	
*E. cuniculi* PCR results			
Positive	6 (75)	12 (66.67)	0.671
Negative	2 (25)	6 (33.33)	
Dazzle test			
Positive	8 (100)	8 (44.44)	0.009 *
Negative	0 (0)	10 (55.56)	

* = Fisher’s exact test was conducted.

**Table 6:** Comparisons of dazzle reflex in *E. cuniculi* seropositive and *E. cuniculi* seronegative rabbits between cataract surgery cases and controls groups.

Group	Dazzle Reflex		*p*-Value
	Positive	Negative	
*E. cuniculi* PCR positive (*n* = 18)			
Cataract surgery case	6 (100)	0 (0)	0.054 *
Control	6 (50)	6 (50)	
*E. cuniculi* PCR negative (*n* = 8)			
Cataract surgery case	2 (100)	0 (0)	0.429 *
Control	2 (33)	4 (66)	

* = Fisher’s exact test was conducted.

## Data Availability

The data presented in this study are available from the corresponding author upon reasonable request.

## Abbreviations

The following abbreviations are used in this manuscript:

OD	Oculus dexter; right eye
OS	Oculus sinister; left eye
OU	Oculus uterque; both eyes
IOL	Intraocular lens
IOP	Intraocular pressures
PCO	Posterior capsular opacities
